# Hospital Saturday Fund

**Published:** 1906-05-05

**Authors:** 


### HOSPITAL SATURDAY FUND.

There was a large attendance at the annual meeting of the Hospital Saturday Fund in the Mansion House on Saturday afternoon, 28th ultimo, the Lord Mayor, Alderman Vaughan Morgan, presiding.

Sir Savile Crossley, the Chairman of the Fund, made a statement as to the work done in 1905. It was, he said, another record year, of which they had had a good many during their 32 years of existence, and he was glad to say they were in a better position than ever. He would like to refer to the efforts continually being made to show that the hospitals were on their last legs, and that the voluntary system was coming to grief. During the week he had seen a letter from a gentleman who said he had been a member of one of the great funds, in which he asserted that in ten years the hospitals would be on the rates. He was quite sure they would not agree with that. (Hear, hear.) In 1897, when the King's Fund was started, the pessimists said that it would ruin the two older funds; but, in fact, they were both getting more money than ever before. Public attention had been called to the work of the hospitals, the standard of excellence had been increased, and so far from the King's Fund having crippled them, both the Saturday and Sunday Funds had progressed more rapidly than ever before. He hoped, therefore, that these pessimistic views would prove equally groundless. Referring to the report of the Council, he said that the abolition of street collections had not led to any loss in the amount collected. The debt on the premises had been further reduced by £368, leaving a balance of £1,131, which they hoped soon to extinguish. The fact that £2,755 was contributed by patients and friends toward the £4,187, which surgical appliances cost, showed that they were justified in asking for public assistance for those who tried to help themselves. The fund continued to be managed with great economy, and their expenses were never so low, being only 9.49 per cent. of the receipts, although they were doing more work than ever. They had helped the Working Classes Sanatorium for Tuberculosis by a large grant, and he hoped that that institution would enable patients to get the necessary treatment while yet there was time to do them good. (Applause.)

Presentations of medallions and diplomas were then made, after which Professor G. Sims Woodhead, M.A., M.D., read a most valuable paper on Alcohol in Relation to Public Health.

The address was received with much applause, after which Mr. Hastings Medhurst moved a vote of thanks to Professor Woodhead, and suggested that the facts set forth in the paper should be taught in the public elementary schools to the children so that they might have a better chance of leading useful and happy lives. Dr. Gambier seconded, and gave it as his experience at Eversfield Hospital that the substitution of milk for alcohol always resulted in a marked change for the better in the patients.

The resolution was carried with applause, and a vote of thanks to the Chairman concluded the proceedings.

